# P-957. Introducing PrEP to Primary Care: A Resident-Focused Initiative

**DOI:** 10.1093/ofid/ofae631.1147

**Published:** 2025-01-29

**Authors:** Hope Oddo Moise, Maren Bell, Danielle Gilbert, Daniel Holmes, Dexter Matrana, Meredith E Clement

**Affiliations:** LSU Health New Orleans, New Orleans, Louisiana; LSUHSC New Orleans, New Orleans, Louisiana; LSU Health New Orleans, Section of Infectious Diseases, New Orleans, Louisiana; Louisiana State University Health Sciences Center, New Orleans, Louisiana; Louisiana State University Health Sciences Center, New Orleans, Louisiana; Louisiana State University Health Science Center–New Orleans, New Orleans, LA

## Abstract

**Background:**

Despite studies showing that HIV pre-exposure prophylaxis (PrEP) dramatically decreases risk of HIV acquisition, it is only currently prescribed to 30% of those who could benefit. The buy in of primary care providers (PCPs), who have an essential role as preventionists, is critical for the most successful reach; however, studies have shown that PCPs often do not feel knowledgeable about PrEP or may be uncomfortable discussing sexual activity or HIV. We hypothesized that dedicated PrEP training for PCPs in a resident clinic would increase prescribing habits.

**Figure:**
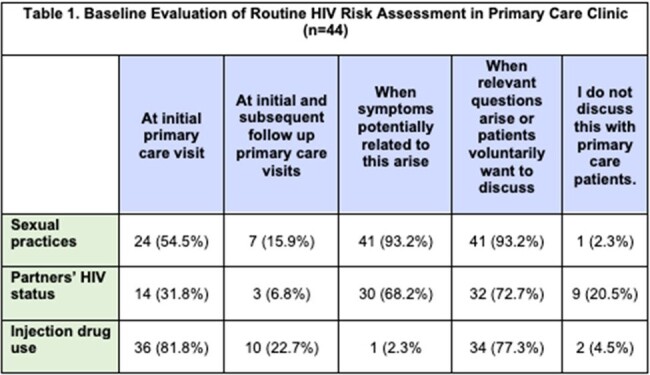

**Methods:**

At an Internal Medicine residency clinic with 73 trainees in New Orleans, LA, a pre-intervention survey was distributed to gauge baseline PrEP knowledge and comfort in discussing sexual practices, HIV status and risk factors. Following the training, residents attended a one-hour PrEP training. A post-training survey then assessed interest and intent to prescribe PrEP. We abstracted primary care clinic records to evaluate PrEP prescribing outcomes post-training, from October 2023 to April 2024.

**Figure:**
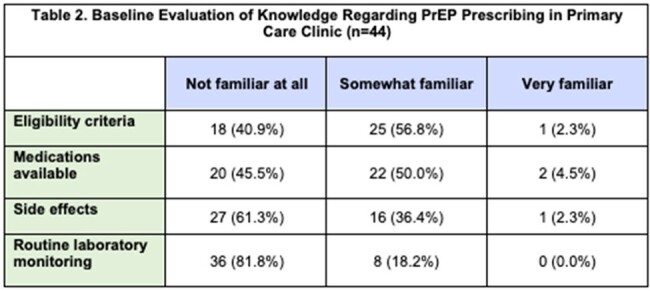

**Results:**

Forty-four residents completed the pre-training assessment. Most indicated non-routine discussions regarding sexual practices (37/44, 84.1%) and HIV risk assessments (41/44, 93.2%). Many trainees reported being not at all familiar with PrEP indications (40.9%), medications (45.5%), or monitoring (81.8%). Most (61.4%) had never discussed PrEP with clients. 53/73 (72.6%) residents attended the educational session, and 26/73 (35.6%) completed the post-training survey. All of the residents who completed both indicated that the training was informative, and 20/21 (95.2%) reported willingness to prescribe PrEP. In the six months post-training, we found that 11 clients were successfully initiated on PrEP. One client was diagnosed with HIV on intake labs, and 3 clients were found to have sexually transmitted infections, all of whom were treated or referred for treatment.

**Figure:**
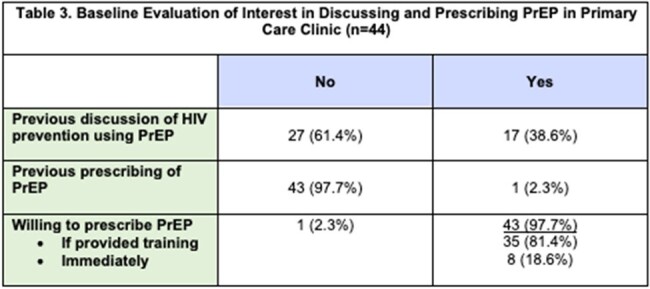

**Conclusion:**

The results of this initiative’s pre-training surveys highlight a lack of routine sexual health and HIV risk assessments in a resident primary care clinic, as well as deficiencies in education regarding HIV PrEP. Following training, residents began prescribing PrEP, demonstrating promise for scaling such educational programs.

PrEP Primary Care Pocket Card

**Figure:**
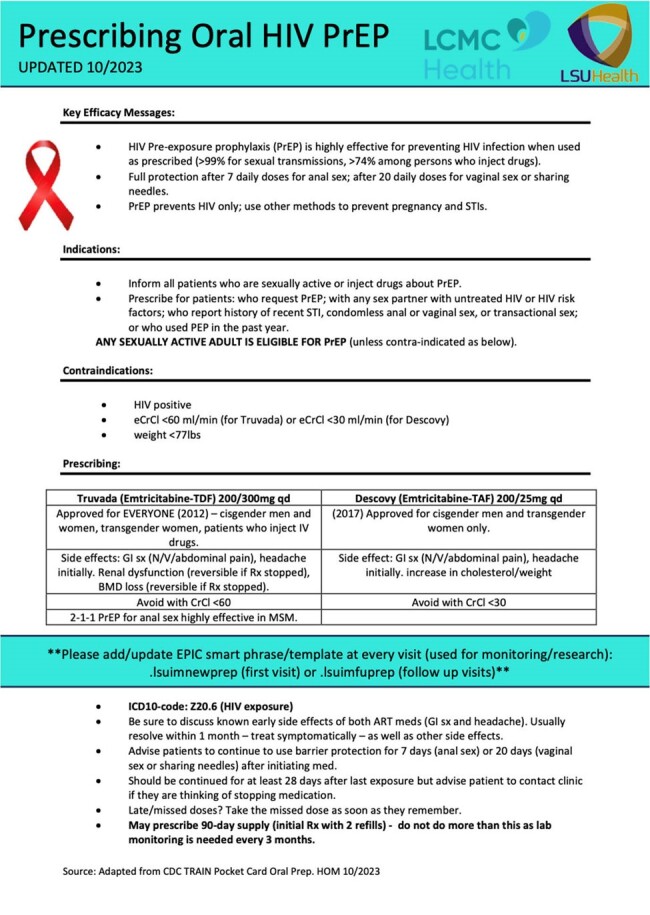

A pocket card adapted from the CDC PrEP TRAIN module was provided to trainees in the resident clinic. Contains information regarding medications available, contraindications, side effects, laboratory monitoring (on back side), ICD-10 code, etc.

**Disclosures:**

**Meredith E. Clement, MD**, Gilead Sciences: Grant/Research Support|Viiv Pharmaceuticals: Advisor/Consultant

